# Prevalent mutator genotype identified in fungal pathogen *Candida glabrata* promotes multi-drug resistance

**DOI:** 10.1038/ncomms11128

**Published:** 2016-03-29

**Authors:** Kelley R. Healey, Yanan Zhao, Winder B. Perez, Shawn R. Lockhart, Jack D. Sobel, Dimitrios Farmakiotis, Dimitrios P. Kontoyiannis, Dominique Sanglard, Saad J. Taj-Aldeen, Barbara D. Alexander, Cristina Jimenez-Ortigosa, Erika Shor, David S. Perlin

**Affiliations:** 1 Public Health Research Institute, New Jersey Medical School, Rutgers Biomedical and Health Sciences, 225 Warren Street, Rutgers, Newark, New Jersey 07103, USA; 2 Centers for Disease Control and Prevention, 1600 Clifton Road, Mailstop G-11, Atlanta, Georgia 30333, USA; 3 Wayne State University School of Medicine, 540 E. Canfield Avenue, 1241 Scott Hall, Detroit, Michigan 48201, USA; 4 The University of Texas MD Anderson Cancer Center, 1400 Pressler Street, FCT12.5046, Unit 1463, Houston, Texas 77030, USA; 5 Warren Alpert Medical School of Brown University, 593 Eddy Street, Gerry House 113, Providence, Rhode Island 02903, USA; 6 Institute of Microbiology of the University Hospital of Lausanne, Rue Bugnon 48, CH-1011 Lausanne, Switzerland; 7 Department of Laboratory Medicine and Pathology, Hamad Medical Corporation, P.O. Box 3050, Doha, Qatar; 8 Duke University, 315 Trent Drive, Hanes House, Room 163A, Durham, North Carolina 27710, USA

## Abstract

The fungal pathogen *Candida glabrata* has emerged as a major health threat since it readily acquires resistance to multiple drug classes, including triazoles and/or echinocandins. Thus far, cellular mechanisms promoting the emergence of resistance to multiple drug classes have not been described in this organism. Here we demonstrate that a mutator phenotype caused by a mismatch repair defect is prevalent in *C. glabrata* clinical isolates. Strains carrying alterations in mismatch repair gene *MSH2* exhibit a higher propensity to breakthrough antifungal treatment *in vitro* and in mouse models of colonization, and are recovered at a high rate (55% of all *C. glabrata* recovered) from patients. This genetic mechanism promotes the acquisition of resistance to multiple antifungals, at least partially explaining the elevated rates of triazole and multi-drug resistance associated with *C. glabrata*. We anticipate that identifying *MSH2* defects in infecting strains may influence the management of patients on antifungal drug therapy.

## Abstract (web summary)

The fungal pathogen *Candida glabrata* readily acquires resistance to multiple types of antifungal drugs. Here, Healey *et al*. show that *C. glabrata* clinical isolates often carry mutations in a gene involved in DNA mismatch repair, and this is associated with increased propensity to develop antifungal resistance.

## Graphical abstract

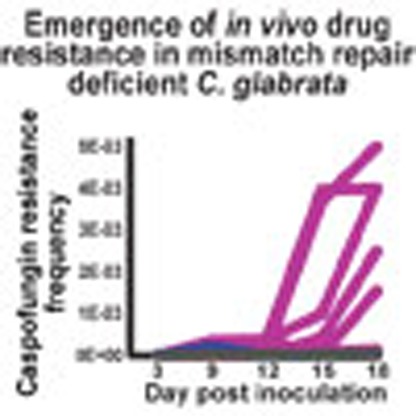

C*andida* species account for the most mucosal and invasive fungal infections worldwide^1^ and are the fourth leading cause of nosocomial bloodstream infections in the United States^2^. *Candida* bloodstream infections cause estimated healthcare costs of $2–4 billion per year in the United States alone and are associated with a crude mortality rate of 40%, even with antifungal treatment^1^. The current arsenal of antifungal drugs is limited, with only two non-toxic antifungal classes to treat candidiasis: triazoles (for example, fluconazole and voriconazole) and echinocandins (for example, caspofungin and micafungin). Polyenes, such as amphotericin B formulations, represent a third class of antifungals, but polyene use is more restricted due to nephrotoxicity^3^. Although 50% of worldwide candidemia cases are caused by *C. albicans*, there has been a steady shift towards non-albicans species over the past 20 years^1^,^4^. In North America, *C. glabrata* now accounts for ∼25% of *Candida* infections^4^,^5^. Albeit this species lacks some important *Candida* virulence factors, including hyphal formation, between 20 and 30% of *C. glabrata* strains exhibit triazole resistance, and this number continues to rise^6^,^7^. *C. glabrata* has also developed resistance to the echinocandins, which were introduced into the market less than 15 years ago, at a faster rate than any other *Candida* species^6^. Depending on the study and geographical location, between 3 and 12% of *C. glabrata* isolates now demonstrate resistance to one or more echinocandin^6^,^8^. Alarmingly, multi-drug resistant (MDR) *C. glabrata*^6^,^8^,^9^,^10^,^11^,^12^, which exhibit resistance to two or more classes of antifungal drugs, are commonly encountered leaving virtually no options for treatment.

Different antifungal drug classes have distinct targets and mechanisms of action and resistance^13^, and it is unclear why *C. glabrata* is capable of rapidly acquiring resistance to multiple drug classes. The acquisition of resistance frequently observed with *C. glabrata* has historically been ascribed to its haploid genome. We hypothesized that a defect in DNA repair may account for accelerated emergence of various genetic changes responsible for drug resistance. Normally, DNA repair is responsible for correcting errors made by DNA polymerase during DNA replication, as well as for repairing DNA damage caused by environmental factors, such as chemicals or radiation, or by endogenous factors, such as reactive oxygen radicals. Thus far, the role of DNA repair in fungal pathogens, especially in the emergence of antifungal resistance, has not been explored in depth.

Here we focused on the role of two DNA repair pathways in *C. glabrata*—mismatch repair (MMR) and double-strand break repair (DSBR)—because defects in these pathways in bacteria^14^ (*in vitro* and *in vivo*) and *C. albicans*^15^ (*in vitro*) have been shown to lead to a hyper-mutable phenotype. More specifically, homozygous disruption of DSBR gene *RAD50* and MMR genes *MSH2* and *PMS1* in *C. albicans* led to an elevated frequency of fluconazole-resistant colonies^15^; therefore, we began our studies examining homologous genes in *C. glabrata*. We found that disruption of *MSH2* in *C. glabrata* led to a hyper-mutable phenotype and significant increases in the emergence of antifungal resistant mutants. Nonsynonymous mutations discovered within *MSH2* in over half (195 out of 357) of the clinical strains analyzed produced similar phenotypes. Our data suggest that defects in mismatch repair represent a key, underlying cellular mechanism that facilitates emergence of resistance to multiple antifungals in *C. glabrata*.

## Results

### *MSH2* deletion leads to an increase in resistant mutants

**Figure 1:**
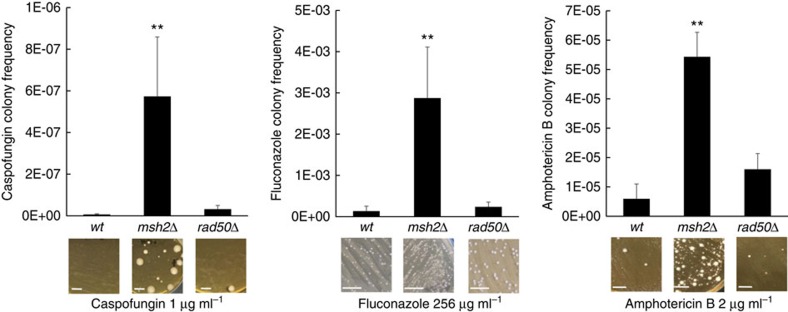
Deletion of *MSH2* in *C. glabrata* leads to significantly more resistant colonies upon selection on multiple antifungal drugs. Wild type, *msh2Δ* and *rad50Δ* strains were selected on media containing caspofungin (an echinocandin), fluconazole (a triazole) and amphotericin B (a polyene) at concentrations from 16- to 32-fold greater than wild type MICs as described in Methods section. The plots show means of resistant colony frequencies from ≥3 independent experiments±s.d. See Supplementary Fig. 1 for resistant frequencies to voriconazole (triazole) and micafungin (echinocandin). **P<0.01 (student’s *t*-test; two-tailed). Representative images of selection plates are shown. Scale bars=1 cm.

To determine the effects of DNA MMR and DSBR on *C. glabrata* antifungal resistance, we separately disrupted one MMR gene, *MSH2* (CAGL0I07733g) and one DSBR gene, *RAD50* (CAGL0J07788g). Echinocandin and fluconazole minimum inhibitory concentrations (MICs) were not significantly changed (<2-fold) in the disruptants when compared with the wild type parental strain (Supplementary Table 1). However, subsequent antifungal selections revealed that the *msh2Δ* strains generated ∼82-, 18- and 9-fold more caspofungin-, fluconazole- and amphotericin B-resistant mutants, respectively, when compared with the wild type strain (Fig. 1). The *rad50Δ* strain did not affect antifungal resistance frequencies as strongly (2 to 5-fold increases relative to wild type). The *msh2Δ* strain also exhibited increased resistance frequencies to micafungin and voriconazole (Supplementary Fig. 1). Over 90% of echinocandin- (caspofungin and micafungin) selected colonies displayed high-level resistance (MICs>2 μg ml^−1^) and 100% of those resistant *msh2Δ* colonies (25/25) displayed a hotspot mutation in either Fks1 or Fks2 (Table 1). Fks1/2 hotspot mutations represent the primary mechanism of echinocandin clinical resistance^16^. *C. glabrata* triazole resistance arises most commonly through overexpression of drug efflux pumps, which is achieved through gain-of-function mutation of the transcription factor Pdr1 (refs 17, 18). As expected, *pdr1* mutations were identified in 10 of 14 fluconazole-resistant wild type and *msh2Δ* mutants (Table 1). Likewise, an *erg6* mutation (Erg6-K157fs) was identified in one amphotericin B-resistant mutant from *msh2Δ* selections (Table 1). Compared with triazoles and echinocandins, molecular mechanisms of polyene resistance are less well described in *C. glabrata*; however mutations in specific *ERG* genes, including *ERG6* have been associated with polyene reduced susceptibility^19^,^20^. These data show that defects in MMR facilitate the emergence of mechanistically diverse *C. glabrata* antifungal drug resistance *in vitro*.

### *msh2* mutations identified in diverse clinical isolates

**Figure 2:**
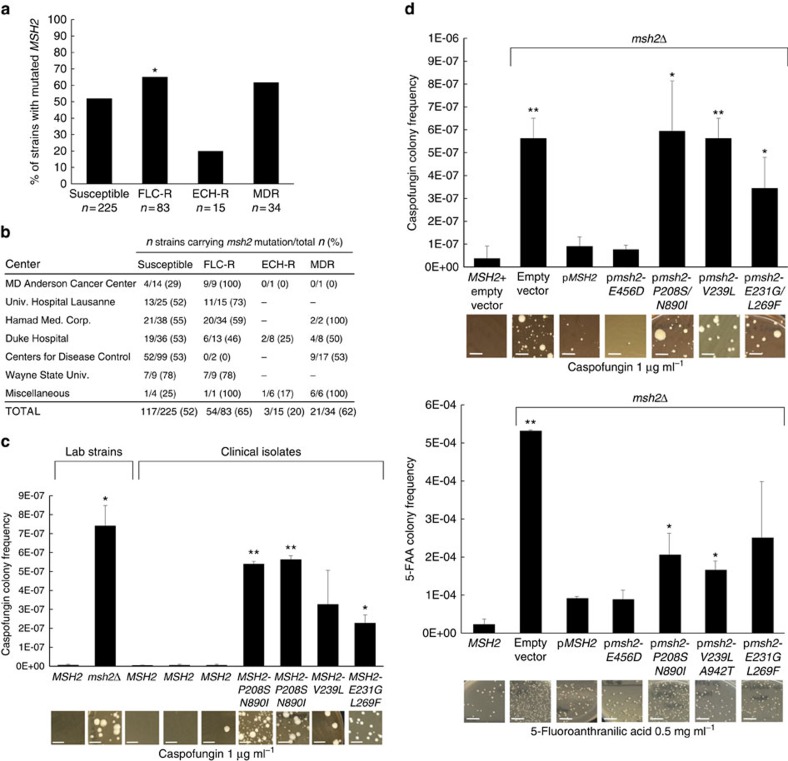
Msh2 alterations identified in diverse clinical isolates cause a mutator phenotype and increased emergence of antifungal resistance. (**a**) The 357 clinical isolates obtained were classified according to their susceptibilities to fluconazole (FLC) and the echinocandins (ECH), and the percentage of isolates within each group demonstrating a nonsynonymous *msh2* mutation were determined. *P* value was determined through *χ*^2^ analysis (compared with susceptible group). (**b**) All isolates were categorized by institution. Isolates demonstrating an *msh2* mutation/total isolates are shown for each susceptibility group. See Supplementary Data 1 for a list of all individual isolates analyzed. (**c**) Echinocandin- (caspofungin) resistant colony frequencies of various clinical isolates were measured. (**d**) Wild type or *msh2Δ* cells expressing an empty or *MSH2*-containing plasmid were selected on caspofungin and 5-fluoroanthranilic acid. See Supplementary Table 4 for strains. Frequency data in **c**,**d** are mean±s.d. from three independent experiments; representative images are shown. *P<0.05, **P<0.01 (student’s *t*-test; two-tailed). Scale bars=1 cm.

To investigate whether defects in MMR contribute to emergence of antifungal resistance in the clinic, we acquired 357 geographically diverse *C. glabrata* strains, including 66 from Duke Hospital (Durham, NC), 118 from the Centers for Disease Control (Atlanta, GA), 25 from MD Anderson Cancer Center (Houston, TX), 18 from Wayne State University Medical School (Detroit, MI), 40 from the University Hospital at Lausanne (Switzerland), 74 from Hamad Medical Corporation (Qatar) and 17 miscellaneous strains from the laboratory collection of one of the authors (D.S.P.). *MSH2* was sequenced in all strains. Overall, 55% of these strains contained a nonsynonymous mutation within Msh2 when compared with the database strain, CBS138/ATCC2001 (http://www.candidagenome.org/). We further assigned these strains into one of four categories: susceptible, fluconazole-resistant (fluconazole^R^), echinocandin-resistant (echinocandin^R^) or MDR (both fluconazole^R^ and echinocandin^R^). Resistant MICs were defined according to the standards recommended by the Clinical and Laboratory Standards Institute (CLSI)^21^,^22^. We do note that although a fluconazole MIC of 32 μg ml^−1^ is defined as susceptible dose dependent, we included these isolates in the fluconazole^R^ group. Seventy-four out of the 81 fluconazole^R^-classified isolates sequenced (all groups except Hamad Medical Corporation) contained a Pdr1 mutation (Supplementary Data 1). Single mutations of specific amino acids in Pdr1, including V329, N768, P927 and T1080, were identified in both clinical isolates and lab-generated mutants (Table 1 and Supplementary Data 1). Echinocandin resistance was declared if an isolate demonstrated a resistant MIC to at least two of the three echinocandin drugs. Forty-five of the 47 echinocandin^R^-classified isolates (echinocandin^R^ and MDR groups) contained an Fks1 or Fks2 hotspot mutation (Supplementary Data 1). Out of the 357 clinical strains analyzed, 52% (117/225) of susceptible isolates demonstrated a nonsynonymous mutation within Msh2, while a mutation was discovered in 65% of fluconazole^R^ isolates (54/83; *P*<0.05 versus susceptible, *χ*^2^=4.1879) and 62% of MDR isolates (21/34; *ns* versus susceptible, *χ*^2^=1.1314) (Fig. 2a,b). We did find individual center differences. For example, strains from Duke Hospital exhibited similar levels of *msh2* mutations in susceptible (53%), fluconazole^R^ (46%) and MDR isolates (50%) (Fig. 2b), whereas isolates obtained from other centers (MD Anderson Cancer Center and University Hospital at Lausanne) demonstrated a pattern consisting of lower levels of mutations in susceptible isolates (29 and 52%) compared with fluconazole^R^ isolates (100 and 73%, both respectively) (Fig. 2b). The only center that provided non-blood stream isolates, Wayne State University Medical School, demonstrated high levels of *msh2* mutations in both susceptible and fluconazole^R^ isolates (78% each). These vaginal isolates were obtained from patients with recurrent vaginitis. We found a low percentage of *msh2* mutations in echinocandin^R^ isolates, although fewer of these isolates were obtained (Fig. 2b). Overall, these data revealed that polymorphisms in the *MSH2* gene are present in an unexpectedly high percentage of clinical *C. glabrata* isolates, particularly fluconazole^R^ strains.

To better understand the timeline of appearance of these *msh2* polymorphisms, we obtained and analyzed paired sets of isolates from the participating centers. Each pair (or triplet) of isolates was isolated from the same patient. Out of these 30 sets of isolates, 20 contained an *msh2* mutation in at least one isolate (Table 2). In all but three sets of isolates that contained an alteration, the identified mutation was present in all isolates from that patient, independent of the susceptibility, suggesting that the *msh2* mutation predated the emergence of fluconazole and/or echinocandin resistance in these patients. Interestingly, in the remaining three sets (all from a single center), we identified differences in Msh2 within isolates taken from the same patient (Table 2). In each case, an *msh2* mutation or additional mutation(s) was only identified within the second, fluconazole^R^ isolate (Table 2).

Notably, 96% of *msh2* mutations in the analyzed clinical isolates contained the following amino acid changes: P208S/N890I, E231G/L269F, V239L/A942T or V239L (see Supplementary Data 1). These amino acids (P208, E231, V239 and L269) map to the protein’s connector region, which is essential for interaction between the MutS (Msh2-Msh3/6) and MutL (Pms1-Mlh1) complexes and therefore, MMR activity^23^. Indeed, upon caspofungin selection *in vitro*, echinocandin susceptible clinical isolates containing these *msh2* mutations produced significantly higher frequencies of echinocandin^R^ colonies and *fks* mutants than isolates carrying wild type *MSH2* (Fig. 2c and Table 1). To directly analyze the functionality of the most commonly identified *msh2* mutations, alleles were amplified from clinical isolates, expressed on a yeast centromere plasmid under the control of their native promoter, and transformed into a laboratory strain carrying a deletion of *MSH2*. Forward mutation frequencies were analyzed by measuring frequencies of colonies resistant to the echinocandin caspofungin (largely due to mutations in *FKS1* or *FKS2*) or to 5-fluoroanthranilic acid (5-FAA; largely due to mutations in *TRP3* or *TRP5*)^24^. As expected, we found that *msh2Δ* exhibited a hyper-mutable phenotype (average caspofungin^R^ colony frequency=5.63 × 10^−7^, average 5-FAA^R^ colony frequency=5.32 × 10^−4^; Fig. 2d). While *MSH2* derived from a wild type strain or from a clinical isolate containing one conservative mutation (E456D) complemented the *msh2Δ* mutant phenotype, expression of either *msh2-P208S/N890I*, *msh2-V239L* or *msh2-E231G/L269F* did not complement *msh2Δ*: the plasmid-borne *msh2* alleles produced the high frequencies of echinocandin^R^ and 5-FAA^R^ colonies characteristic of the *msh2Δ* mutant (Fig. 2d). The resulting echinocandin^R^ colonies contained Fks mutations, specifically *fks1-S629P*, *fks2-S663P*, *fks2-S663Y* and *fks2-S663F* (Table 1), all of which have been previously shown to lead to echinocandin treatment failure^25^,^26^,^27^. Also, ∼25% of the 5-FAA^R^ colonies contained *trp3* nonsynonymous mutations (Supplementary Table 2). Together, these data suggest that clinical strains of *C. glabrata* carry mutations in *MSH2*, which result in MMR defects and promote the emergence of antifungal resistance.

### Colonization with *msh2Δ* leads to increased resistance *in vivo*

**Figure 3:**
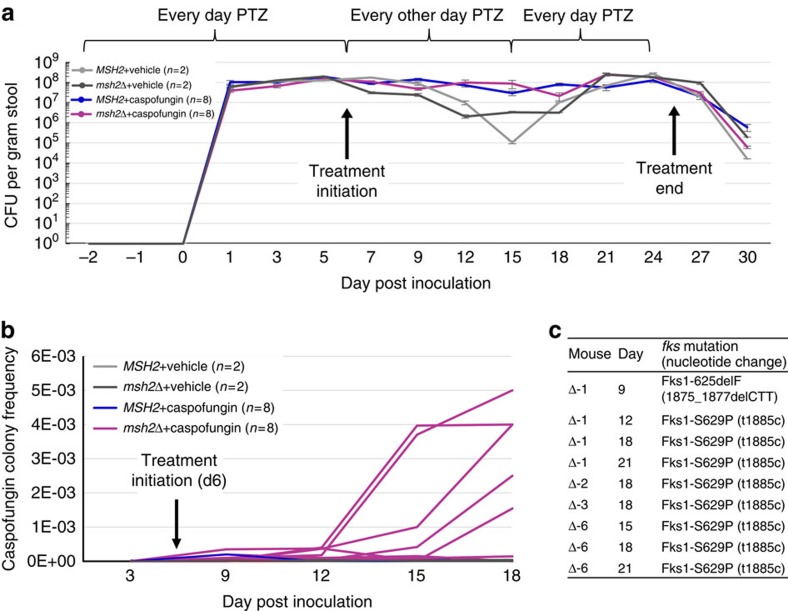
Colonization with *msh2Δ* leads to increased echinocandin-resistance *in vivo*. (**a**) GI colonization of CF-1 mice represented through fecal *C. glabrata* burdens. Daily administration (i.p.) of caspofungin (CSF; 0.5 mg ml^−1^) or saline occurred from days 6 to 26 post-inoculation. (CSF-treated groups: *n*=8 wild type inoculated mice, 8 *msh2Δ* inoculated mice; Saline-treated groups: *n*=2 wild type, 2 *msh2Δ*). Means±s.d. are calculated. (**b**) Fecal samples from each mouse at days 3, 9, 12, 15 and 18 (−3, 3, 6, 9 and 12 days post-treatment initiation, respectively) were selected on caspofungin (2 μg ml^−1^) containing YPD media (see Methods section for additional details). Five of eight CSF-treated mice that were inoculated with *msh2Δ* demonstrated increase in resistant colony frequencies between days 12 and 18. (**c**) *fks1* mutants identified within fecal isolates. Colonies on antifungal-free plates (produced in part **a**) were replica plated onto caspofungin-containing media. Hotspot regions of *FKS1* and *FKS2* (echinocandin drug targets) were amplified and sequenced in resistant colonies.

To determine if altered MMR activity can promote the development of *C. glabrata* antifungal resistance *in vivo*, we utilized a mouse model of *C. glabrata* gastrointestinal (GI) colonization (Fig. 3a). This model recapitulates pathogenesis of human disease through overgrowth of yeast in the primary reservoir (GI). First, bacterial flora were eradicated from the GI tract with a daily piperacillin-tazobactam (PTZ) regimen^28^ (Fig. 3a). Mice were then orally inoculated with either a wild type or *msh2Δ* strain followed by daily, sub-inhibitory echinocandin (caspofungin; 0.5 mg kg^−1^ i.p.) or vehicle (saline i.p.) administration for 21 days. Mice were effectively colonized (range: 5 × 10^7^ to 1 × 10^8^ CFU per gram of stool) with both the wild type and *msh2Δ* strains from days 1 to 27 post-inoculation (Fig. 3a). Of note, no significant growth difference between these strains *in vitro* was observed (Supplementary Fig. 2). Caspofungin resistant colony frequencies from each mouse were tracked at one time point prior to and four time points following treatment initiation over a period of 18 days. While we observed no increases in resistant yeast recovered from eight caspofungin-treated mice that were colonized with the wild type strain, five of eight caspofungin-treated mice that were colonized with the *msh2Δ* strain demonstrated an increase in resistant colonies 6 to 12 days following treatment initiation, representative of an expanding *fks* mutant population (Fig. 3b). DNA sequencing revealed that echinocandin resistance was due to *fks1* mutations in yeast recovered from those mice (Fig. 3c). This suggests that loss of MMR in *C. glabrata* can lead to an increased frequency of resistance-conferring mutations *in vivo*.

### Fitness of *msh2Δ* assayed in mixed inoculation mouse models

**Figure 4:**
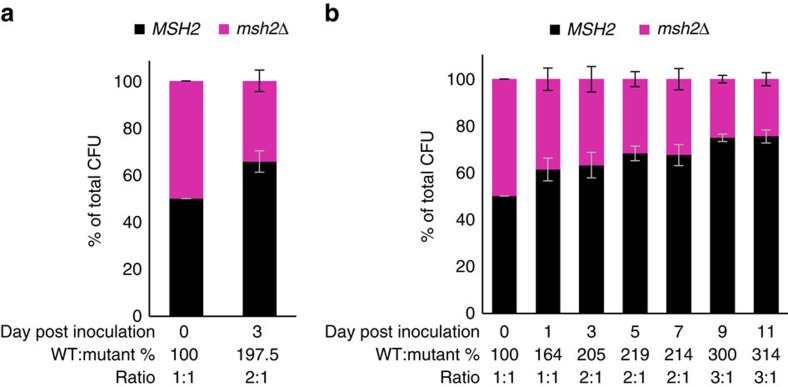
Wild type cells partially outcompete *msh2Δ* in mixed inoculation mouse models. DNA was isolated from kidney tissue of systemically infected, immunosuppressed mice (**a**) and from fecal samples of GI-colonized, immune competent mice (**b**) and then subjected to SYBR green quantitative PCR with strain specific primers. Wild type (WT) to mutant ratios were calculated from qPCR DNA copy numbers. Means±s.d. are calculated.

In patients, strains containing defects in MMR may coexist with strains with wild type MMR function. Therefore, we performed fitness assays using both the aforementioned GI colonization model, as well as a systemic infection mouse model to assess the fitness of a MMR-deficient strain *in vivo* (see Methods section for details). Mice were inoculated with either wild type, *msh2Δ*, or an equal CFU mix of both strains. In the systemic model, kidneys were harvested at day 3 post-infection and subject to CFU plating and qPCR analysis following DNA isolation. Specific primers were designed for both wild type and *msh2Δ* strains (Supplementary Table 3). SYBR green real-time PCR was used to quantify DNA copy number and calculate strain ratios. Similarly, fecal samples collected from colonized mice at day 1, 3, 5, 7, 9 and 11 post-inoculation were assessed for mixed population ratio. In the systemic infection model, the average wild type to mutant ratio found in kidneys isolated from mice infected with both strains was 2:1 (Fig. 4a), indicating a modest fitness defect of the *msh2Δ* strain. Similarly, we found an increasing wild type to mutant ratio in fecal samples over the course of the colonization model (Fig. 4b). Importantly, both models demonstrated similar levels of colonization and infection when inoculated with either wild type or *msh2Δ* alone (Supplementary Fig. 3).

## Discussion

While antifungal drug resistance is nearly always due to fungal genome alterations ranging from point mutations to gain or loss of whole chromosomes^13^, the identity of specific DNA repair pathways that impinge on the emergence of *C. glabrata* resistance has remained unknown. Here, we have shown that an unexpectedly high percentage (55%) of clinical *C. glabrata* isolates carry loss-of-function mutations in the MMR gene *MSH2*, which produced a mutator phenotype resulting in accelerated emergence of antifungal drug resistance both *in vitro* and *in vivo*. Interestingly, these mutations were more frequent among fluconazole^R^ and MDR *C. glabrata* isolates (Fig. 2a), suggesting that their appearance may be associated with fluconazole exposure. While *msh2* mutations were also found in nearly half of all drug susceptible isolates, we must emphasize that many patients were severely ill and had most likely been exposed to multiple drugs, including antibiotics, steroids, antineoplastics and/or antifungals, which may have also influenced the frequency of *msh2* mutations. Differences in patient populations and antifungal administration practices are likely responsible for the significant discrepancies observed in the frequencies of *msh2* mutations in susceptible and resistant isolates across different institutions (Fig. 2a,b).

The lower percentage of *msh2* mutations identified in echinocandin^R^ clinical isolates may be due to a small number of such strains available for analysis. Alternatively, we cannot exclude a negative genetic interaction between defects in MMR and *fks1/2* mutations, resulting in a fitness defect. Fks1-S629P was the primary mutation identified from the stool of echinocandin-treated mice colonized with *msh2Δ* (Fig. 3c), and the most frequent *fks1* mutation among *C. glabrata* MDR clinical isolates with MMR defects (Supplementary Data 1). Whether this mutation is associated with MDR and/or influences *C. glabrata* fitness remains to be investigated. The equivalent *C. glabrata* mutation in Fks2 (S663P) was recently shown to limit the macrophage inflammatory response upon caspofungin exposure, suggesting certain *fks* mutations have a higher propensity for immune evasion^29^, similar to *pdr1* mutations^30^.

The observation that only a limited spectrum of *msh2* mutations were recovered from a large and diverse patient population may indicate that these mutations did not arise *de novo* in different patients, but that they pre-existed and were enriched for during drug treatment or pathogenesis (that is conversion from colonizer to infectious strain), perhaps by conferring a selective advantage under those conditions. This hypothesis is consistent with reports indicating that a mutator phenotype may be advantageous for fungal microevolution and survival within the host^31^. Also, the scenario where *C. glabrata* strains defective in MMR coexist with wild type strains in the host is consistent with our observation that the *msh2Δ* strain had only a mild fitness disadvantage *in vivo* when present together with the wild type strain (Fig. 4). This fitness defect may result from the accumulation of mutations in essential genes; however, in single challenge studies, the *msh2Δ* strain exhibited comparable or slightly higher levels of colonization and infection relative to wild type (Supplementary Fig. 3). Indeed, given that over half of our patient samples, including nearly half of all susceptible isolates, contain *msh2* loss-of-function mutations, we suspect *msh2* mutant colonizing strains have adapted well to their host, possibly though acquisition of gain-of-fitness mutations (for example, *pdr1* mutation^32^).

Previously described fungal mechanisms of genetic instability that generate genetic diversity under stress have primarily involved gross chromosomal rearrangements and/or aneuploidy^33^. In contrast, our study presents the first example of a clinical, fungal population carrying a mutator phenotype due to genetic defects in a specific DNA repair pathway, namely MMR. It is possible that this mechanism of genetic instability is specific to *C. glabrata* because, unlike the other prevalent *Candida* species (*C. albicans*, *C. parapsilosis*, *C. tropicalis* and *C. krusei*), *C. glabrata* is a haploid organism where a single DNA repair mutation is sufficient to cause an associated mutator phenotype and subsequent emergence of resistance-conferring mutation(s). We suggest that the elevated levels of both triazole and multi-drug resistance associated with *C. glabrata* are at least partially due to the presence of *msh2* mutations. Based on our results, by the time a patient requires administration of an antifungal, there is a substantial chance that the infecting strain contains an *msh2* mutation. Therefore, we propose that *C. glabrata MSH2* may be used as a biomarker prior to and throughout the course of therapy to determine the propensity of any given *C. glabrata* isolate to generate drug-resistant mutants and break through antifungal treatment. Thus, our study may have implications for antifungal prophylaxis, recurrent and long-term therapies, and antifungal stewardship protocols.

## Methods

### Ethics statement

Mice were housed in Public Health Research Institute’s Animal Biosafety Level-2 Research Animal Facility (ICPH RAF), a center of the New Jersey Medical School, Rutgers University (NJMS-Rutgers). Our animal facility follows the Public Health Service and National Institute of Health Policy of Humane Care and Use of Laboratory Animals. All experimental protocols were approved by the Rutgers Institutional Animal Care and Use Committee (IACUC).

### Strains and media

A total of 357 *C. glabrata* clinical strains were obtained from patients with *C. glabrata* bloodstream or vaginal infections, including 66 from Duke Hospital (Durham, NC), 118 from the Centers for Disease Control (Atlanta, GA, USA), 25 from MD Anderson Cancer Center (Houston, TX, USA), 18 from Wayne State University Medical School (Detroit, MI, USA), 40 from the University Hospital at Lausanne (Switzerland), 74 from Hamad Medical Corporation (Qatar) and 17 additional miscellaneous strains from the laboratory collection of one of the authors (D.S.P.). Miscellaneous strains were obtained from the American Type Culture Collection (ATCC), Seongmin Lee (University of Texas), Hector Bonilla (Summa Health Systems), Barbara Alexander/Rachel Addison (Duke University), Marion Tuohy (Cleveland Clinic) and Soo-Hyun Kim (Chonnam National University Medical School) (Supplementary Data 1). Entire or partial MIC and/or *Fks* mutational data from Duke University^6^, CDC^11^ and S.H. Kim^12^ (‘miscellaneous’ group) isolates have been previously published. The University Hospital at Lausanne isolates were all collected between the years of 1993 and 1996, predating echinocandin clinical use. Hotspots of the *FKS1* and *FKS2* genes were sequenced in echinocandin-resistant isolates. *PDR1* was sequenced in fluconazole-resistant and select fluconazole-sensitive isolates from each collection except the Hamad Medical Corporation isolates. *MSH2* was sequenced in all isolates. See Supplementary Table 3 for primers. Plasmid pGRB2.0 was obtained from Brendan Cormack (John’s Hopkins University School of Medicine)^34^. Yeast strains were maintained in YPD (1% yeast extract, 2% peptone and 2% dextrose) or synthetically-defined media lacking uracil (SD-ura) or tryptophan (SD-trp) (dropout base plus complete supplement mixture minus uracil or tryptophan). Plasmid-bearing DH5α *E. coli* strain was maintained in 40 μg ml^−1^ ampicillin-supplemented Luria-Bertani (LB) media. Plasmid-bearing *C. glabrata* strains (Supplementary Table 4) were maintained in SD-ura media.

### Gene disruption and cloning

*C. glabrata* ATCC 200989 (2001 HTU) or 2001 HTL (ref. 35) (gift from Karl Kuchler, University of Vienna) was transformed with a PCR-amplified *TRP1* deletion cassette (see Supplementary Table 3 for primers). The deletion primers contain flanking regions that are homologous to the immediate up- and down-stream regions of each target gene’s coding region. Transformed cells were selected on synthetically-defined media lacking tryptophan, and colonies were PCR-screened and sequenced to confirm disruption. For plasmid-based expression of *MSH2*, a gap-repair approach was used^36^. Briefly, coding regions plus promoter regions were PCR-amplified from laboratory or clinical isolates with primers (Supplementary Table 3) containing flanking regions homologous to regions upstream and downstream of the SmaI restriction site in pGRB2.0. Strain 2001 HTU or *msh2Δ* were transformed with this PCR product, along with SmaI linearized pGRB2.0, and selected on SD-ura media. The resulting strains are listed in Supplementary Table 4.

### Drug susceptibility assays

Fluconazole and echinocandin minimum inhibitory concentrations (MICs) were determined by broth microdilution following CLSI standards as described^21^,^22^, except for the Qatar isolates, which were analyzed via Etest methodology according to the manufacturer’s instructions (Liofilchem, Italy). Fluconazole (LKT Laboratories, Saint Paul, MN), caspofungin (Merck, Rahway, NJ) and micafungin (Astellas, Deerfield, IL) were dissolved and diluted according to CLSI standards^21^. Fluconazole resistance was declared if an isolate demonstrated a CLSI-defined susceptible dose-dependent (32 μg ml^−1^) or resistant (≥64 μg ml^−1^) MIC, while echinocandin resistance was declared if an isolate demonstrated CLSI-defined resistance to at least two of the three echinocandins (≥0.5 μg ml^−1^ for caspofungin and anidulafungin, ≥0.25 μg ml^−1^ for micafungin).

### Drug selections and mutant analysis

Strains were grown overnight in appropriate media (YPD or SD-ura) at 37 °C. We aimed to plate ∼1 × 10^8^ CFU onto echinocandin-containing drug plates and between 1 × 10^5^ and 1 × 10^6^ CFU onto fluconazole, voriconazole (Pfizer), amphotericin B (Sigma-Aldrich, Milwaukee, WI) and 5-fluoroanthranillic acid (5-FAA, Sigma-Aldrich) plates. Drug concentrations of mutant selection plates were 16- to 32-fold greater than the wild type strain MICs. 5-FAA media was prepared as previously described^24^. A minimum of three biological replicate selections per strain were performed. Dilutions were plated onto drug-free media to determine exact CFU counts. Frequencies were calculated as number of colonies on the drug plate divided by the total CFU plated. Frequency averages were calculated from at least three independent selections from the concentration(s) indicated in Table 1. Colonies were spotted onto plates containing two-fold higher drug concentrations and then appropriate target genes, for example, *FKS1* hotspot 1, *FKS2* hotspot 1, *PDR1*, *ERG6* and *TRP5*, were PCR-amplified and sequenced in resistant colonies (see Supplementary Table 3 for primers).

### Colonization mouse model

Six-week-old female CF-1 immunocompetent mice (Charles River Laboratories) were treated (s.c.) from day -2 to day 6 with 320 mg kg^−1^ PTZ(8:1) to clear native intestinal bacterial flora^28^. PTZ was then injected every other day (days 8, 10, 12, 14, 16) and returned to daily injections near the end of the experiment (days 17–25). On day 0, mice were inoculated via oral gavage with ∼1.5 × 10^8^ CFU of *C. glabrata* 2001 HTL wild type or isogenic strain *mshΔ* (Supplementary Table 4) in 100 μl of saline. Strain 2001 HTL was utilized instead of 2001 HTU (ATCC 200989) because the histidine, leucine and tryptophan auxotrophies do not significantly affect the fitness or virulence of *C. glabrata*^35^. Fresh fecal samples were collected throughout the experiment to assess fungal GI tract colonization. Fungal burden was determined by plating proper dilutions of fecal samples onto drug-free YPD plates and recorded as *C. glabrata* CFU per gram of stool. Daily administration of caspofungin (0.5 mg kg^−1^) or saline (100 ul i.p.) was initiated on day 6 post inoculation, when stabilized colonization was observed, and continued through day 26. Colonies on burden plates were replica-plated onto caspofungin (2 μg ml^−1^) plates and *FKS1* and *FKS2* hotspots were PCR-amplified and sequenced in those demonstrating resistance. Caspofungin-resistant colony frequencies were determined through selection of fecal samples on YPD plates supplemented with caspofungin (2 μg ml^−1^), PTZ (16 μg ml^−1^) and chloramphenicol (20 μg ml^−1^). The increased caspofungin resistant colony frequencies observed with the animal fecal selections are approximately a 3.7 log increase (5000 × ) from the caspofungin selection of the untreated *msh2Δ* strain (compare Fig. 1 with Fig. 3a), allowing us to conclude that the expanded *fks* mutant populations within these mice account for that frequency difference. Nonetheless, replica-plating from drug-free plates was used to uncover *fks* mutants to ensure the mutant arose in the animal as opposed to on the plate.

### Fitness assays

For the colonization fitness study, mice were colonized as above, but with an equal mix of *C. glabrata* 2001 HTL wild type and isogenic strain *mshΔ* totaling 1.5 × 10^8^ CFU in 100 μl of saline. Control inoculum groups were colonized with 1.5 × 10^8^ CFU of a single strain. PTZ treatment was administered daily from day -2 to day 11. Fecal samples were collected on days 0, 1, 3, 5, 7, 9 and 11. Plating of fecal material and CFU counts were performed as stated above. For DNA extraction, approximately half of the homogenized fecal pellet was subjected to a 2 h incubation with proteinase K (50 μg ml^−1^; Ambion) followed by purification with QIAamp DNA Mini Kit (Qiagen). DNA was isolated from the pellets of each mouse at each time point. DNA was then subjected to SYBR green quantitative PCR (qPCR) using strain-specific primers (Supplementary Table 3) to determine copy number of each strain within the samples. SYBR green qPCR was performed on a Mx3005P real-time instrument (Agilent) using Rotor-Gene SYBR Green PCR kit (Qiagen). The thermal cycling conditions consisted of an initial activation step at 95 °C for 5 min and 35 cycles at 95 °C for 10 s, and 60 °C for 20 s, followed by a dissociation step at 95 °C for 1 min, ramping temperature from 55 to 95 °C at the rate of 0.2 °C s^−1^. Specificity of the primers was confirmed by observing a single peak in the dissociation curve. The detection limit and amplification efficiency of both assays were evaluated by testing 10-fold diluted DNA series (10 ng∼100 fg per reaction) for wild type, *msh2Δ*, 10:1 mix of wild type and *msh2Δ*, 1:1 mix of wild type and *msh2Δ*, and 1:10 mix of wild type and *msh2Δ*. The 1:1 mix of wild type and *msh2Δ* were also used as external standards for strain quantification in each animal sample testing run.

For the systemic infection study, neutropenia was induced in 8-week-old BALB/c mice (Charles River Laboratories) through administration of 150 mg kg^−1^ cyclophosphamide i.p. on day -4 and 100 mg kg^−1^ on days -1 and day 2 post infection. Mice were challenged (i.v.) with an equal mix of *C. glabrata* 2001 HTL wild type and *msh2Δ* totaling 4.0 × 10^7^ CFU in 100 μl of saline. Control groups were infected with 4.0 × 10^7^ CFU of a single strain. On day 3 post infection, mice were euthanized and kidneys harvested. Half of each kidney was homogenized and dilutions plated for total CFU counts and the remaining halves were subjected to DNA isolation as stated above, except with an overnight proteinase K digestion. DNA was subjected to the same qPCR analysis as the fecal DNAs.

### Statistics

All data analyses were performed using GraphPad Prism, version 5.0, software for Windows (GraphPad Software, San Diego, CA). *χ*^2^ analysis was used to determine differences in *msh2* mutational status between susceptibility groups. Differences of resistant frequency between strains were evaluated by Student’s *t*-test. A *P* value of <0.05 (two-tailed) is considered statistically significant.

## Additional information

**How to cite this article:** Healey, K. R. *et al*. Prevalent mutator genotype identified in fungal pathogen *Candida glabrata* promotes multi-drug resistance. *Nat. Commun.* 7:11128 doi: 10.1038/ncomms11128 (2016).

## Supplementary Material

Supplementary Information Supplementary Figures 1-3, Supplementary Tables 1-4 and Supplementary Reference

## Figures and Tables

**Table 1:**
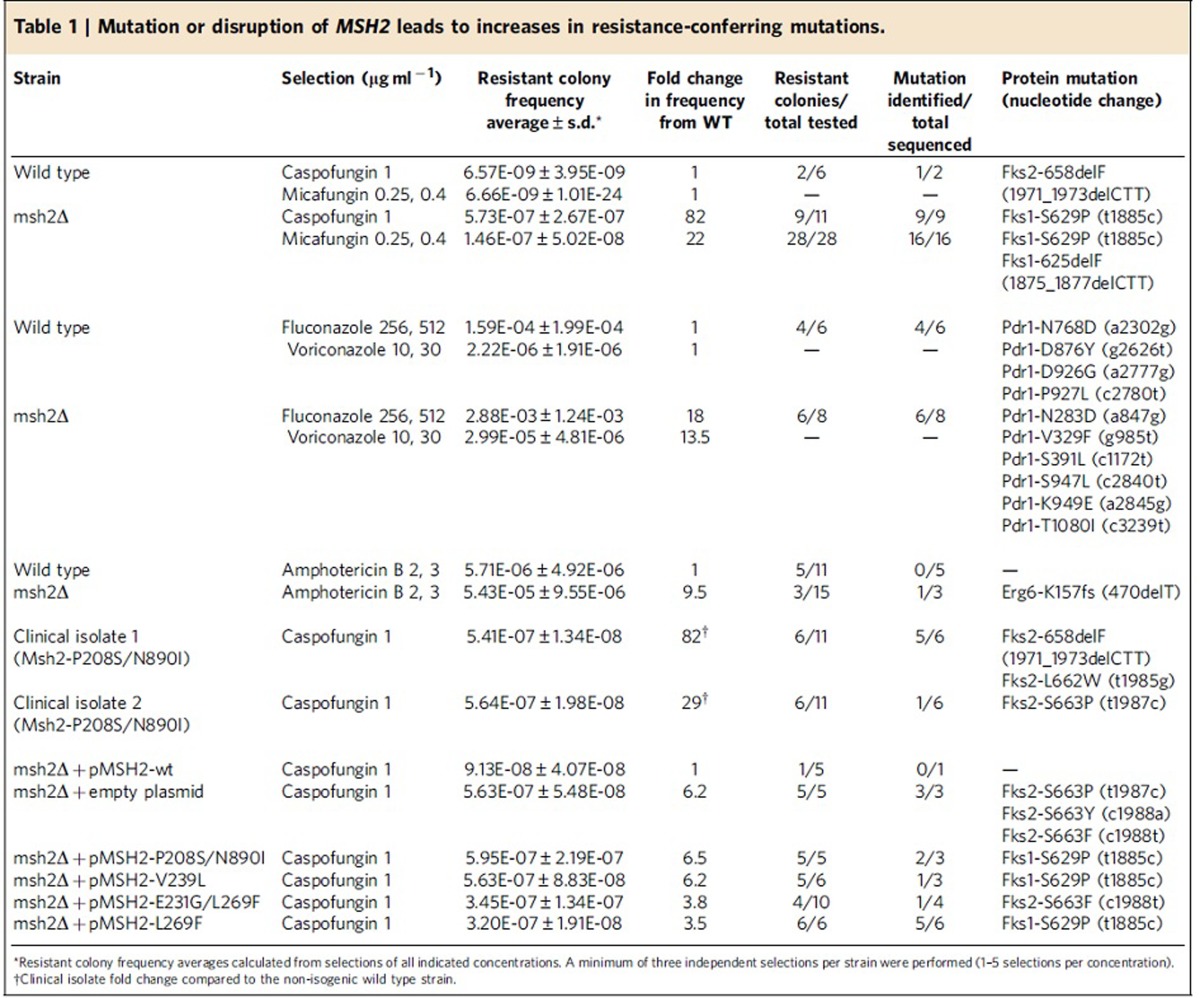
Mutation or disruption of *MSH2* leads to increases in resistance-conferring mutations.

**Table 2:** Paired patient isolates containing *msh2* mutations.

Strain*	Susceptibility	Msh2	Pdr1 gain-of-function†	Fks1/2 hotspots
UHL 2270	Susceptible	Wild type	Wild type	—
UHL 2271	FLC-R	V239L	D261G	—
				
UHL 2256	Susceptible	E456D‡	Wild type	—
UHL 2257	FLC-R	V239L	N691D	—
				
UHL 2247	Susceptible	V239L	G493D	—
UHL 2248	FLC-R	V239L/K814E/A942T	E1083Q	—
				
UHL 2267	Susceptible	V239S/A942T	-	—
UHL 2266	Susceptible	V239S/A942T	Wild type	—
UHL 2268	FLC-R	V239S/A942T	S316I	—
				
UHL 2272	Susceptible	V239L/A942T	Wild type	—
UHL 2273	FLC-R	V239L/A942T	R293I	—
				
UHL 2276	Susceptible	V239L/A942T	Wild type	—
UHL 2277	FLC-R	V239L/A942T	R592S	—
				
UHL 2278	Susceptible	V239L/A942T	Wild type	—
UHL 2279	FLC-R	V239L/A942T	G583S	—
				
UHL 529	Susceptible	V239L/A942T	Wild type	—
UHL 530	FLC-R	V239L/A942T	E1083Q	—
				
UHL 753	Susceptible	V239L/A942T	Wild type	—
UHL 2311	Susceptible	V239L/A942T	Wild type	—
UHL 2312	FLC-R	V239L/A942T	Y584C	—
				
UHL 2280	Susceptible	V239L/A942T	—	—
UHL 2281	Susceptible	V239L/A942T	—	—
UHL 2282	FLC-R	V239L/A942T	—	—
				
UHL 2298	Susceptible	V239L/A942T	—	—
UHL 2299	Susceptible	V239L/A942T	—	—
				
WSU 162-11	FLC-R	E231G/L269F	R295G	—
WSU 659-11	FLC-R	E231G/L269F	R265G	—
				
WSU 86-14	FLC-R	E231G/L269F	E340G	—
WSU 339-14	Susceptible	E231G/L269F	E340G	—
				
WSU 599-02	Susceptible	V239L	Wild type	—
WSU 906-05	FLC-R	V239L	D1082G	—
				
WSU 961-00	FLC-R	V239L/A942T	R792stop	—
WSU 057-02	FLC-R	V239L/A942T	R792stop	—
				
WSU 880-03	Susceptible	E231G/L269F	—	—
WSU 847-10	Susceptible	E231G/L269F	—	—
				
WSU 1080-10	Susceptible	Q259H	—	—
WSU 584-14	Susceptible	Q259H	—	—
				
Duke 299	Susceptible	E231G/L269F	Wild type	Wild type
Duke 307	ECH-R	E231G/L269F	Wild type	Fks2-S663P
				
Duke 246	FLC-R	P208S/N890I	G1079R	Wild type
Duke 249	MDR	P208S/N890I	G1079R	Fks2-S663P
				
SK 1	FLC-R	V239L	L825P	Wild type
SK 2	MDR	V239L	L825P	Fks2-I660_L661insF

^*^UHL: University Hospital at Lausanne, WSU: Wayne State University, Duke: Duke University, SK: gift of Soo-Hyun Kim (Miscellaneous group).

^†^Common polymorphisms identified in both sensitive and resistant isolates not included.

^‡^E456D does not significantly alter *MSH2* activity (see Fig. 2).

## References

[b1] Pfaller M. A. & Diekema D. J. Epidemiology of invasive mycoses in North America. Crit. Rev. Microbiol. 36, 1–53 (2010). 20088682 10.3109/10408410903241444

[b2] Wisplinghoff H. . Nosocomial bloodstream infections in US hospitals: analysis of 24,179 cases from a prospective nationwide surveillance study. Clin. Infect. Dis. 39, 309–317 (2004). 15306996 10.1086/421946

[b3] Sabra R. & Branch R. A. Amphotericin B nephrotoxicity. Drug Saf. 5, 94–108 (1990). 2182052 10.2165/00002018-199005020-00003

[b4] Diekema D., Arbefeville S., Boyken L., Kroeger J. & Pfaller M. The changing epidemiology of healthcare-associated candidemia over three decades. Diagn. Microbiol. Infect. Dis. 73, 45–48 (2012). 22578938 10.1016/j.diagmicrobio.2012.02.001

[b5] Pfaller M. A., Moet G. J., Messer S. A., Jones R. N. & Castanheira M. Candida bloodstream infections: comparison of species distributions and antifungal resistance patterns in community-onset and nosocomial isolates in the SENTRY Antimicrobial Surveillance Program, 2008–2009. Antimicrob. Agents Chemother. 55, 561–566 (2011). 21115790 10.1128/AAC.01079-10 PMC3028787

[b6] Alexander B. D. . Increasing echinocandin resistance in Candida glabrata: clinical failure correlates with presence of FKS mutations and elevated minimum inhibitory concentrations. Clin. Infect. Dis. 56, 1724–1732 (2013). 23487382 10.1093/cid/cit136 PMC3658363

[b7] Pfaller M. A. & Diekema D. J. Epidemiology of invasive candidiasis: a persistent public health problem. Clin. Microbiol. Rev. 20, 133–163 (2007). 17223626 10.1128/CMR.00029-06 PMC1797637

[b8] Pfaller M. A. . Frequency of decreased susceptibility and resistance to echinocandins among fluconazole-resistant bloodstream isolates of Candida glabrata. J. Clin. Microbiol. 50, 1199–1203 (2012). 22278842 10.1128/JCM.06112-11 PMC3318516

[b9] Farmakiotis D., Tarrand J. J. & Kontoyiannis D. P. Drug-resistant *Candida glabrata* infection in cancer patients. Emerg. Infect. Dis. 20, 1833–1840 (2014). 25340258 10.3201/eid2011.140685 PMC4214312

[b10] Bizerra F. C. . Breakthrough candidemia due to multidrug-resistant *Candida glabrata* during prophylaxis with a low dose of micafungin. Antimicrob. Agents Chemother. 58, 2438–2440 (2014). 24468776 10.1128/AAC.02189-13 PMC4023795

[b11] Pham C. D. . Role of FKS mutations in *Candida glabrata*: MIC values, echinocandin resistance, and multidrug resistance. Antimicrob. Agents Chemother. 58, 4690–4696 (2014). 24890592 10.1128/AAC.03255-14 PMC4136002

[b12] Cho E. J. . Emergence of multiple resistance profiles involving azoles, echinocandins and amphotericin B in Candida glabrata isolates from a neutropenia patient with prolonged fungaemia. J. Antimicrob. Chemother. 70, 1268–1270 (2015). 25550394 10.1093/jac/dku518

[b13] Cowen L. E., Sanglard D., Howard S. J., Rogers P. D. & Perlin D. S. Mechanisms of antifungal drug resistance. Cold Spring Harb. Perspect. Med. 5, a019752 (2014). 25384768 10.1101/cshperspect.a019752 PMC4484955

[b14] Chopra I., O'Neill A. J. & Miller K. The role of mutators in the emergence of antibiotic-resistant bacteria. Drug. Resist. Updat 6, 137–145 (2003). 12860461 10.1016/s1368-7646(03)00041-4

[b15] Legrand M., Chan C. L., Jauert P. A. & Kirkpatrick D. T. Role of DNA mismatch repair and double-strand break repair in genome stability and antifungal drug resistance in *Candida albicans*. Eukaryot. Cell 6, 2194–2205 (2007). 17965250 10.1128/EC.00299-07 PMC2168241

[b16] Perlin D. S. Mechanisms of echinocandin antifungal drug resistance. Ann. NY. Acad. Sci. 1354, 1–11 (2015). 26190298 10.1111/nyas.12831 PMC4626328

[b17] Vermitsky J. P. . Pdr1 regulates multidrug resistance in *Candida glabrata*: gene disruption and genome-wide expression studies. Mol. Microbiol. 61, 704–722 (2006). 16803598 10.1111/j.1365-2958.2006.05235.x

[b18] Tsai H. F., Krol A. A., Sarti K. E. & Bennett J. E. *Candida glabrata* PDR1, a transcriptional regulator of a pleiotropic drug resistance network, mediates azole resistance in clinical isolates and petite mutants. Antimicrob. Agents Chemother. 50, 1384–1392 (2006). 16569856 10.1128/AAC.50.4.1384-1392.2006 PMC1426987

[b19] Vandeputte P. . Reduced susceptibility to polyenes associated with a missense mutation in the ERG6 gene in a clinical isolate of *Candida glabrata* with pseudohyphal growth. Antimicrob. Agents Chemother. 51, 982–990 (2007). 17158937 10.1128/AAC.01510-06 PMC1803144

[b20] Vandeputte P. . A nonsense mutation in the ERG6 gene leads to reduced susceptibility to polyenes in a clinical isolate of *Candida glabrata*. Antimicrob. Agents Chemother. 52, 3701–3709 (2008). 18694952 10.1128/AAC.00423-08 PMC2565872

[b21] National Commitee for Clinical Laboratory Standards. Reference method for broth dilution antifungal susceptibility testing of yeasts; approved standard 3rd ed Vol. 28, Clinical and Laboratory Standards Institute document M27-S4 (2012).

[b22] Pfaller M. A. & Diekema D. J. Progress in antifungal susceptibility testing of Candida spp. by use of Clinical and Laboratory Standards Institute broth microdilution methods, 2010–2012. J. Clin. Microbiol. 50, 2846–2856 (2012). 22740712 10.1128/JCM.00937-12 PMC3421803

[b23] Mendillo M. L. . A conserved MutS homolog connector domain interface interacts with MutL homologs. Proc. Natl Acad. Sci. USA 106, 22223–22228 (2009). 20080788 10.1073/pnas.0912250106 PMC2796910

[b24] Briones-Martin-del-Campo M. . The superoxide dismutases of *Candida glabrata* protect against oxidative damage and are required for lysine biosynthesis, DNA integrity and chronological life survival. Microbiology 161, 300–310 (2015). 25479837 10.1099/mic.0.000006

[b25] Arendrup M. C. . Differential in vivo activities of anidulafungin, caspofungin, and micafungin against *Candida glabrata* isolates with and without FKS resistance mutations. Antimicrob. Agents Chemother. 56, 2435–2442 (2012). 22354305 10.1128/AAC.06369-11 PMC3346593

[b26] Garcia-Effron G., Lee S., Park S., Cleary J. D. & Perlin D. S. Effect of Candida glabrata FKS1 and FKS2 mutations on echinocandin sensitivity and kinetics of 1,3-beta-D-glucan synthase: implication for the existing susceptibility breakpoint. Antimicrob. Agents Chemother. 53, 3690–3699 (2009). 19546367 10.1128/AAC.00443-09 PMC2737881

[b27] Perlin D. S. Echinocandin resistance, susceptibility testing and prophylaxis: implications for patient management. Drugs 74, 1573–1585 (2014). 25255923 10.1007/s40265-014-0286-5 PMC4201113

[b28] Pultz N. J., Stiefel U., Ghannoum M., Helfand M. S. & Donskey C. J. Effect of parenteral antibiotic administration on establishment of intestinal colonization by *Candida glabrata* in adult mice. Antimicrob. Agents Chemother. 49, 438–440 (2005). 15616330 10.1128/AAC.49.1.438-440.2005 PMC538875

[b29] Beyda N. D., Liao G., Endres B. T., Lewis R. E. & Garey K. W. Innate inflammatory response and immunopharmacologic activity of micafungin, caspofungin, and voriconazole against wild-type and FKS mutant *Candida glabrata* isolates. Antimicrob. Agents Chemother. 59, 5405–5412 (2015). 26100700 10.1128/AAC.00624-15 PMC4538525

[b30] Vale-Silva L., Ischer F., Leibundgut-Landmann S. & Sanglard D. Gain-of-function mutations in PDR1, a regulator of antifungal drug resistance in *Candida glabrata*, control adherence to host cells. Infect. Immun. 81, 1709–1720 (2013). 23460523 10.1128/IAI.00074-13 PMC3648025

[b31] Magditch D. A., Liu T. B., Xue C. & Idnurm A. DNA mutations mediate microevolution between host-adapted forms of the pathogenic fungus *Cryptococcus neoformans*. PLoS Pathog. 8, e1002936 (2012). 23055925 10.1371/journal.ppat.1002936 PMC3464208

[b32] Ferrari S., Sanguinetti M., Torelli R., Posteraro B. & Sanglard D. Contribution of CgPDR1-regulated genes in enhanced virulence of azole-resistant *Candida glabrata*. PLoS ONE 6, e17589 (2011). 21408004 10.1371/journal.pone.0017589 PMC3052359

[b33] Shapiro R. S. Antimicrobial-induced DNA damage and genomic instability in microbial pathogens. PLoS Pathog. 11, e1004678 (2015). 25811381 10.1371/journal.ppat.1004678 PMC4374783

[b34] Zordan R. E. . Expression plasmids for use in *Candida glabrata*. G3 (Bethesda) 3, 1675–1686 (2013). 23934995 10.1534/g3.113.006908 PMC3789792

[b35] Jacobsen I. D. . Candida glabrata persistence in mice does not depend on host immunosuppression and is unaffected by fungal amino acid auxotrophy. Infect. Immun. 78, 1066–1077 (2010). 20008535 10.1128/IAI.01244-09 PMC2825948

[b36] Healey K. R., Katiyar S. K., Raj S. & Edlind T. D. CRS-MIS in *Candida glabrata*: sphingolipids modulate echinocandin-Fks interaction. Mol. Microbiol. 86, 303–313 (2012). 22909030 10.1111/j.1365-2958.2012.08194.x PMC3472958

