# Supplementary material for: Prevalent mutator genotype identified in fungal pathogen Candida glabrata promotes multi-drug resistance
Source: Nat Commun. 2016 Mar 29;7:11128. doi: 10.1038/ncomms11128 (PMC5603725; doi:10.1038/ncomms11128)
Supplement: Supplementary Information — Supplementary Figures 1-3, Supplementary Tables 1-4 and Supplementary Reference [file ncomms11128-s1.pdf]

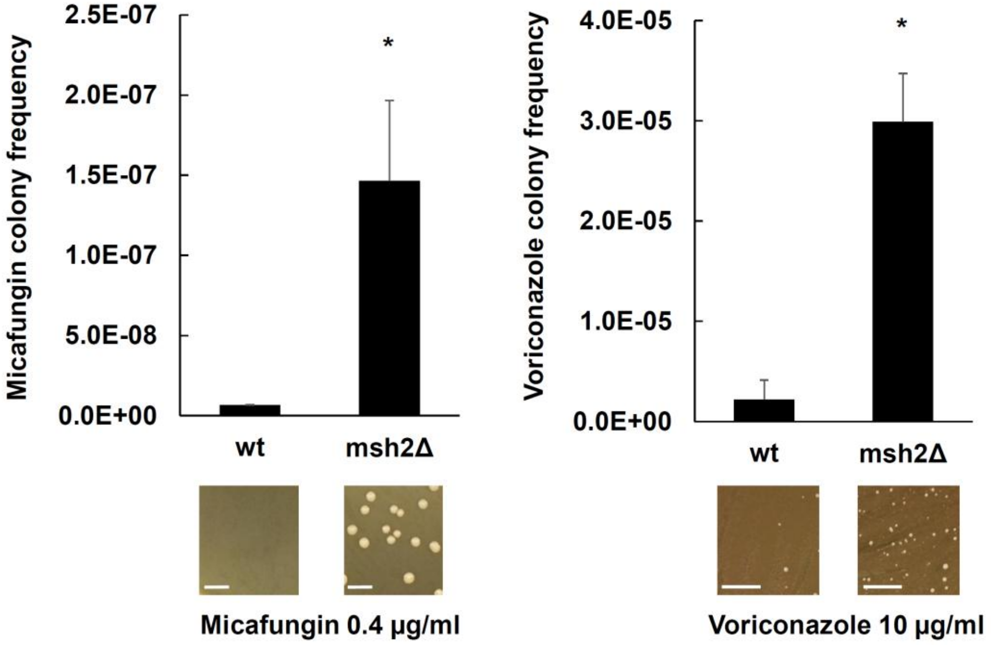

Supplementary Figure 1. ***C. glabrata msh2Δ*** **leads to increased micafungin- and voriconazole-resistant colonies.**

Wild type and *msh2Δ* strains were selected on media containing micafungin (an echinocandin) and voriconazole (a triazole) at concentrations 16- to 32-fold greater than wild type MICs as described in Methods. Plots show means of resistant colony frequencies from ≥ 3 independent experiments ± s.d. *$P < 0.05$, **$P < 0.01$ (student's t-test; two-tailed). Representative images of selection plates are shown. Scale bars = 1 cm.

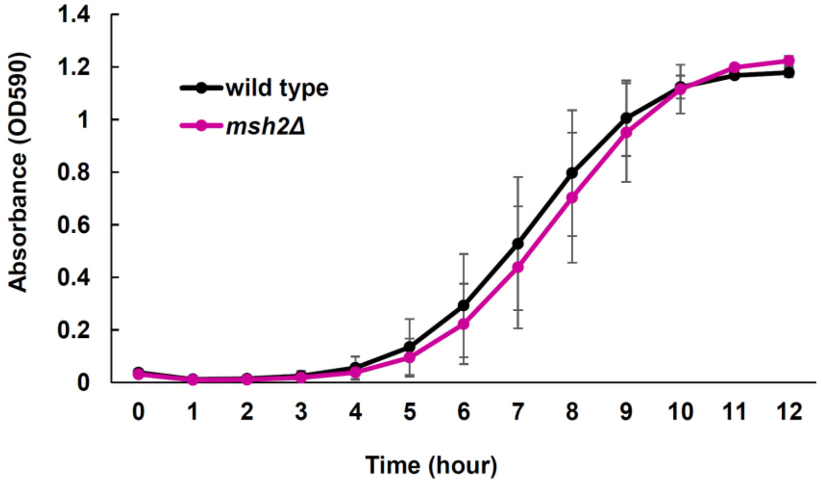

Supplementary Figure 2. No growth difference observed *in vitro* between *C. glabrata* wild type and *msh2Δ*.

Wild type and *msh2Δ* strains (2001 HTL strain background) were incubated in YPD media overnight and then diluted to an optical density (OD590nm) of 0.1. Cells were grown at 37 degrees Celsius and absorbance values read each hour. The curve represents the mean of three independent experiments ± s.d.

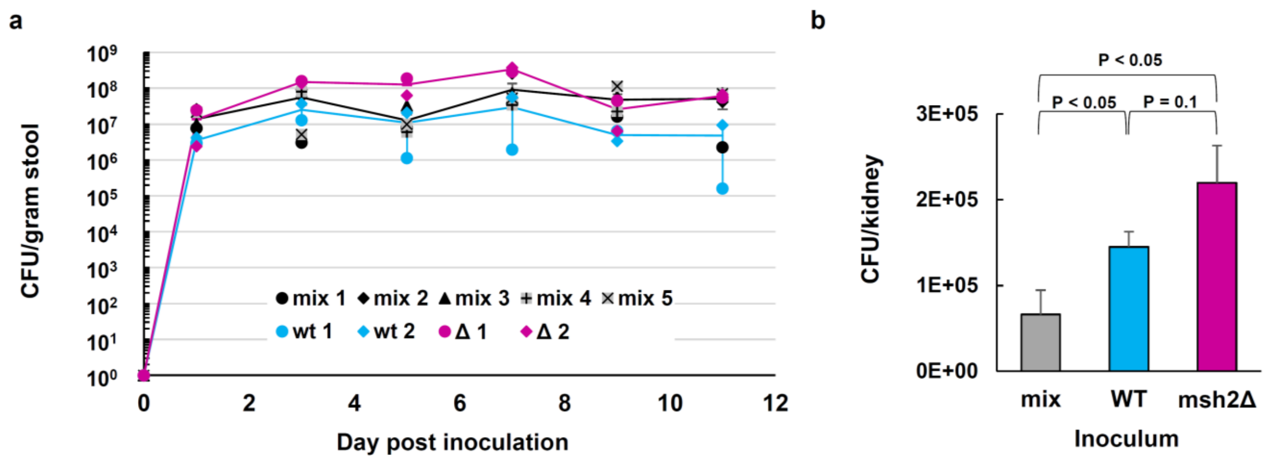

Supplementary Figure 3. *C. glabrata* wild type and *msh2Δ* strains colonize the GI tract and systemically infect mice to similar levels.

Fitness assayed though both GI colonization (**a**) and systemic infection (**b**) mouse models (see Methods for details). **a,** Immune competent CF-1 mice were colonized as in **Fig. 3a**. Fecal samples were collected on the indicated days and dilutions plated for CFU counts. Burdens measured in each mouse are indicated with color-coded lines connecting average group CFU counts. **b,** Immunosuppressed BALB/c mice were injected (i.v.) with wild type, *msh2Δ*, or equal CFU from both strains. Kidneys were harvested three days post infection and burden levels determined after plating appropriate dilutions. **a, b,** Means ± s.d. are calculated.

Supplementary Table 1. *C. glabrata msh2Δ* and *rad50Δ* MICs.

	MIC (µg ml^{-1})		
C. glabrata strain	Fluconazole	Caspofungin	Micafungin
Wild type	16	0.06	0.06
msh2Δ	16	0.06	0.12
rad50Δ	16	0.03	0.06

Supplementary Table 2. 5-FAA colony frequencies and identified mutations.

Strain	Colony Frequency	Fold change in frequency from WT	Mutation identified/ total sequenced	Protein mutation (nucleotide change)
Wild type (ATCC 2001)	2.34×10^{-5}	1	3/15	Trp3-W311stop (c933t)<br>Trp3-V4A (t11c)<br>Trp3-G292R (g874a)
msh2Δ + empty plasmid	5.32×10^{-4}	23	1/7	Trp3-Q169stop (c505t)
msh2Δ + *pMSH2*	9.21×10^{-5}	4	-	-
msh2Δ + *pmsh2-P208S/N890I*	2.06×10^{-4}	9	2/5	Trp3-E392G (t1175c)<br>Trp3-G411S (c1231t)
msh2Δ + *pmsh2-V239L*	1.67×10^{-4}	7	1/5	Trp3-S444Y (g1331t)

Supplementary Table 3. Primers used in this study

Primer*	Application	Sequence (5'-3')†
CgMSH2-TRP1F	*msh2Δ*	TGCTAAGTATAAATTACTCCAACAAACGTTTGTGCTATTTATTGTTGGAATCAAGAAGACAGAGAAAATGTCTGTTATTAATTTCACAGG
CgMSH2-TRP1R	*msh2Δ*	TTCCGGAAAAAATTATTTAAGCTTTACATAACATTCCCGTTATCAACAGTTTAAAAACTGCGTTGTACTATTTCTT AGCATTTTTGACGA
CgMSH2c543R	screen	GTTCGAGTAAGCCTCATTGTC
CgMSH2u273F	screen, PCR	CGATGAGCCGATCACTTTAC
CgMSH2c846R	sequence	ACCTAGCATCTCTTGTTCAC
CgMSH2c1626R	sequence	CCTGGTCAATCTCATACACC
CgMSH2c2340R	sequence	CCATGCAAGTCCAAAACCATC
CgMSH2d145R	PCR, sequence	ACTTATCGAGGCACTCAGTC
pGRB-MSH2F	gap-repair	TCGAGGTCGACGGTATCGATAAGCTTGATATCGAATTCCGAAGAAGGCCAGTTCAAACTC
pGRB-MSH2R	gap-repair	GAGCTCCACCGCGGTGGCGGCCGCTCTAGAACTAGTGGATACTTATCGAGGCACTCAGTC
T7	screen	GCGTAATACGACTCACTATAGG
T3	screen	AAGCGCGCAATTAACCCTCAC
ScTRP1c417R	disruption screen	CGAATGAGGTTTCTGTGAAGC
CgRAD50-TRP1F	*rad50Δ*	AGCTAGGTAATATGGAGGGAAAAGTGACATATACTATTCGAGAAACTAGTGCAGGGATATATGACTTATGTCTGTT ATTAATTTCACAGG
CgRAD50-TRP1R	*rad50Δ*	CAAAATTTAGTGCGGCCCGGTTGTAGCTCAATGTAAAAACATCTTGGCTAGTAGCAGGTTTTTCATCCTATTTCTT AGCATTTTTGACGA
CgRAD50u180F	screen	CATCTCACCCTCTCGTTGTA
CgRAD50c500R	screen	TCACTCAGTGGCCATAGACT
CgFKS1c1757F	PCR	ACGTCGCTTCTCAAACCTTC
CgFKS1c2225R	PCR, sequence	GCGTTCCAGACTTGGGAAAT
CgFKS2c1790F	PCR	CGATTATGCCATTAGGTGGTC
CgFKS2c2165R	PCR, sequence	CCAACAGAGAAGACAGTGTTGA
CgPDR1u175F	PCR	AACAAGCATAGAGGCGCTGT
CgPDR1c828R	sequence	AAGTGACTTAGTGGTGGCAC
CgPDR1c1703R	sequence	GCAACAGCTACATTCAAGACC
CgPDR1c2600R	sequence	CTGCATACTTTGGCACTCT
CgPDR1d110R	PCR, sequence	TGAGGTAGTCTAAGTCTCATG
CgERG6u159F	PCR	AGTTATCGGGTAAACATCGC
CgERG6c520R	sequence	CGAAATCCATGTGGTCTTGC
CgERG6d101R	PCR, sequence	GACAGGTAACACTCATAAGGC
CgTRP3u121F	PCR	GCATTTCAGGAGTAGTCAGTC
CgTRP3d50R	PCR	ACATAGTGTCTAGAAGTGCTC
CgTRP3c758R	sequence	ATTTGGCTTGGTGCTAGACC
CgTRP3c612F	sequence	CACCTCAACTGGCAATAACC
MSH2WT-F130	qPCR	ACTGCTGTTGGTGATGATGC
MSH2WT-R284	qPCR	AACAAACTAGCCACGATCTGC
MSH2delta-F193	qPCR	AATAGTTCAGGCACTCCGAA
MSH2delta-R416	qPCR	GAATGAGGTTTCTGTGAAGC

*****Numbers in primer names correspond to nucleotide location upstream (u) or within the coding region (c) relative to the start codon, or downstream (d) relative to the stop codon.
†Underlined regions of deletion primers correspond to *S. cerevisiae TRP1* coding sequences; underlined regions of gap-repair cloning primers correspond to *C. glabrata MSH2* upstream or downstream sequences, and non-underlined regions correspond to sequences on pGRB2.0 surrounding SmaI restriction site.

Supplementary Table 4. Strains produced in this study

Strain	Source
200989/2001 HTU (2001 *ura3*, *trp1Δ*, *his1Δ*)	ATCC*
Δrad50::*TRP1*	this study
Δmsh2::*TRP1*	this study
Δmsh2 + pGRB2.0	this study
Δmsh2 + pGRB2.0-*MSH2* (wild type)	this study
Δmsh2 + pGRB2.0-*msh2 P208S/N890I*	this study
Δmsh2 + pGRB2.0-*msh2 E231G/L269F*	this study
Δmsh2 + pGRB2.0-*msh2 L269F*	this study
Δmsh2 + pGRB2.0-*msh2 V239L/A942T*	this study
Δmsh2 + pGRB2.0-*msh2 V239L*	this study
Δmsh2 + pGRB2.0-*msh2 E456D*	this study
200989 + pGRB2.0	this study
2001/CBS138	ATCC
2001 HTL (*his-*, *trp-*, *leu-)*	K. Kuchler[1]
Δmsh2::*TRP1*	this study

*American Type Culture Collection (Manassas, VA)

Supplementary references

1 Jacobsen, I. D. *et al.* Candida glabrata persistence in mice does not depend on host immunosuppression and is unaffected by fungal amino acid auxotrophy. *Infect Immun* **78**, 1066-1077, doi:10.1128/IAI.01244-09 (2010).
